# Percutaneous Vertebroplasty Relieves Pain in Cervical Spine Metastases

**DOI:** 10.1155/2017/3926318

**Published:** 2017-01-23

**Authors:** Li Bao, Pu Jia, Jinjun Li, Hao Chen, Yipeng Dong, Fei Feng, He Yang, Mengmeng Chen, Hai Tang

**Affiliations:** Department of Orthopedics, Beijing Friendship Hospital, Capital Medical University, Beijing, China

## Abstract

Percutaneous vertebroplasty (PVP) has been shown to release spinal pain and stabilize the vertebral body. PVP is suggested as an alternative treatment in spinal metastasis. Although cervical metastases is less prevalent than thoracic and lumbar spine, PVP procedure in cervical vertebrae remains technical challenging. We retrospectively analyzed the data from patients (*n* = 9) who underwent PVP using anterolateral approach to treat severe neck pain and restricted cervical mobility from metastatic disease. Patients were rated using modified Tokuhashi score and Tomita score before the procedure. Visual analog scale (VAS), neck disability index (NDI), analgesic use, and imaging (X-ray or CT) were evaluated before PVP and 3 days, 3 months, and 6 months after PVP. All patients were in late stage of cancer evaluated using modified Tokuhashi and Tomita score. The cement leakage rate was 63.6% (14 of the 22 vertebrae) with no severe complications. VAS, NDI, and analgesic use were significantly decreased 3 days after the procedure and remained at low level until 6 months of follow-up. Our result suggested PVP effectively released the pain from patients with cervical metastasis. The results warrant further clinical investigation.

## 1. Introduction

Percutaneous vertebroplasty (PVP) was first reported by Galibert et al. to treat vertebral angioma in 1987 [1]. Over the past decades, PVP has been well developed with extensive usage in cases for pain relief and bone strengthening of the vertebral body [2]. Spinal metastasis is among the most occurring sites of skeletal metastases [3]. The most common targets of spinal metastasis are thoracic vertebrae (60%–80%), followed by lumbar (20%) and cervical spine (10%) [4]. Surgery and radiotherapy have been the main treatment options for those patients; however, they result in a number of complications. PVP was recently suggested as an alternative treatment for spinal metastatic patients who find surgery and radiotherapy intolerable. PVP procedure includes percutaneous injection of polymethyl methacrylate (PMMA) into a vertebral body. Although PVP provided pain control and vertebral stabilization in most cases [5, 6], PVP application in the cervical spine remains technically challenging in part due to complex anatomy of this region. The procedure can be performed using multiple approaches; however, only a few reports described the procedures via anterolateral approach. In this report, we evaluated the technical feasibility, efficacy, and complications in PVP using anterolateral approach in late-stage cancer patients with multiple cervical spinal metastases.

## 2. Materials and Methods

### 2.1. Patients

Patients were recruited between July 2009 and September 2014 in Beijing Friendship Hospital, who underwent PVP to treat severe neck pain and restricted cervical mobility from metastatic disease. All patients were in late stage of cancer and were refractory to radiotherapy or chemotherapy. The patients were evaluated before the procedure with a multidisciplinary approach including thorough interview and physical examination, excluding coagulation disorders, systemic infection, nerve root type pain, neurological deficits, and spinal surgery contraindications. X-ray and computed tomography (CT) were performed to evaluate integrity of the vertebral body and to confirm multiple cervical metastases (≥2). Written informed consent was obtained from each patient before the PVP.

### 2.2. Procedure

**Figure 1:**
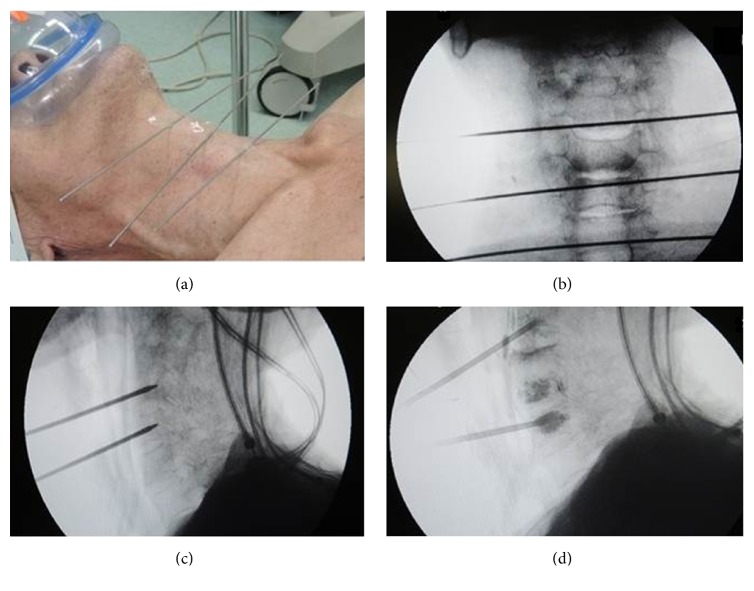
(a) PVP was performed using anterolateral approach with all patients in supine position under general anesthesia or local anesthesia. (b) The target vertebra was located under the guidance of a C-arm digitalized X-ray machine. (c) Confirm the corrected position of the needle. (d) The injection was stopped when the cement filled up the lesion or any paraspinal leakage was observed.

The procedures were performed with the patients in supine position under general anesthesia or local anesthesia (Figure 1(a)). Anterolateral approach was performed for all patients using precision cement delivery system (Stryker, MI, USA) and the target vertebra was located under the guidance of a C-arm digitalized X-ray machine (Figures 1(a) and 1(b)). The skin was incised below the angle of the mandible, paralleling of trachea and esophagus at the targeted side, followed by incision of subcutaneous tissue and fascia layer. Kirschner needle was advanced through the interior side of sternocleidomastoid muscle into the anteroinferior surface of the targeted vertebra. After confirming the corrected position of the needle under C-arm digitalized X-ray machine (Figure 1(c)), the needle was injected into the middle of vertebra body and replaced by a guiding needle. Working channel was inserted along the guiding needle followed by slow injection of SpinePlex Bone Cement that has been prepared as “toothpaste” stage. The injection condition was checked using C-arm digitalized X-ray machine when every 0.5 mL volume of the cement was injected and was stopped when the cement filled up the lesion or any paraspinal leakage was observed (Figure 1(d)). The volume of the injected cement was determined using a graduated syringe.

### 2.3. Assessment Index

Modified Tokuhashi scoring system [7] (score from 0–15) is used to evaluate the prognosis of metastatic spinal tumors and selected treatment methods. A score between 0 and 8 indicates a predicted survival period less than 6 months with a suggestion of conservative or palliative treatment, while 9–11 and 12–15 correspond to a predicted survival period of more than 6 months and 1 year, respectively. Tomita scoring system [8] (score from 2–10) is used to guide treatment strategy for spinal metastases. Specifically, 2-3 points suggested a wide or marginal excision with a predicted long-term survival; 4-5 points suggested marginal or intralesional excision with a predicted middle-term survival; 6-7 points suggested palliative surgery with a predicted short-term survival; and 8–10 points indicated nonoperative supportive care. Visual analog scale [9] (VAS) is used to measure pain with a score ranging from 0 (absence of pain) to 10 (maximum pain). Neck disability index [10] (NDI) is an instrument used to assess neck pain related disability. Specifically, 0–20% suggests normal or minimal disability, 20–40% suggests mild disability, 40–60% suggests moderate disability, 60–80% suggests severe disability, and 80% or over suggests complete disability. Analgesic use was scored from 0 to 4. A score of 0 indicates none, 1 indicates use of nonsteroid anti-inflammatory drugs, 2 indicates oral narcotics as needed, 3 indicates oral narcotics as scheduled, and 4 indicates parenteral narcotics.

All patients were rated using modified Tokuhashi score and Tomita score before the procedure. VAS, NDI, and analgesic use were evaluated before PVP and 3 days, 3 months, and 6 months after PVP. Imaging was performed at 3 days, 3 months, and 6 months using X-ray or CT to examine the cement leakage.

### 2.4. Statistical Analysis

Data are presented as mean ± standard deviation. The results of VAS, NDI, and analgesic use at all time points were analyzed using ANOVA with SPSS statistical software, version 17.0. The threshold for statistical significance was set at *P* < 0.05.

## 3. Results

**Figure 2:**
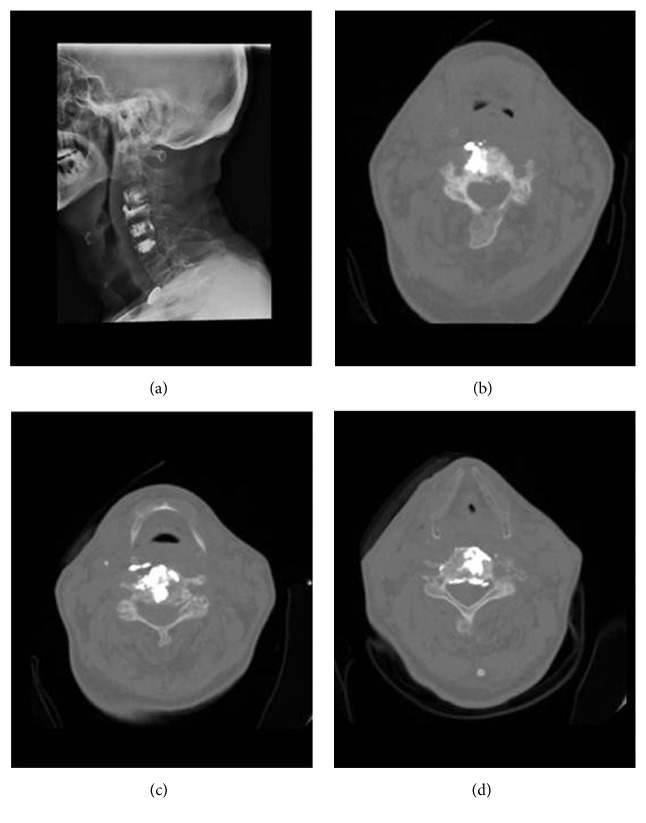
(a) CT image of one patient after PVP was performed in 4 cervical spines. Cement leakage was observed at the right side of (b) C3 and (c) C4 and in the spinal canal of (d) C5.

There was 9 patients (6 men and 3 women with a median age of 57, ranging from 47 to 75) with a total number of 22 cervical vertebrae who underwent PVP using anterolateral approach between July 2009 and September 2014 in Beijing Friendship Hospital. All patients were at the late stage of cancer, indicated by the mean of modified Tokuhashi score (6.89 ± 2.14) and Tomita score (7.56 ± 1.13). Detailed baseline demographics and disease characteristics were shown in Table 1. The mean of injected cement volume was 1.32 ± 0.49 ml. The cement leakage rate was 63.6% (14 of the 22 vertebrae) (Figure 2). No serious complication was observed. One patient had numbness in arms which disappeared after neurotrophic treatment. One patient developed long-term mild numbness in arms that could not be relieved by neurotrophic treatment. Three patients had pain when swallowing after PVP but self-recovered after a short period. Two patients died during 6-month follow-up period: one died from cervical paraplegia in the fourth month, and the other died from multiple organ failure in the fifth month.

VAS score decreased from 8.11 ± 1.45 to 2.22 ± 0.67 at 3 days after PVP, significantly released the pain of the patients (*P* < 0.001). Importantly, VAS remained at low level throughout the 6 months of follow-up period (Table 2). Similarly, both postoperative analgesic score and NDI reduced significantly compared to preoperative condition and remained at lower level during the follow-up period (both *P* < 0.01, Table 2).

## 4. Discussion

In this report, we retrospectively analyzed the data from patients with multiple cervical spinal metastases who underwent PVP using an anterolateral approach. Conservative or palliative treatment is suggested in patients with limited predicted survival period. In our study, all patients were evaluated using modified Tokuhashi score and Tomita score before the procedure, indicating a late-stage metastatic cancer. PVP has been shown to release spinal pain and stabilize vertebral body. In particular, PVP has been suggested in the treatment of metastatic vertebral fracture [11]. We found that PVP significantly released the pain from our patients with few complications, consistent with previous study using PVP to treat metastasis in cervical spine [12, 13]. Specifically, our patients showed significantly reduced VAS, NDI, and analgesic score examined from 3 days after the operation until 6 months of follow-up time. Several possible mechanisms have been proposed of PVP in treating metastatic vertebrae, including the following: (1) The filled cement indirectly blocks tumor blood supply; (2) increased internal temperature during cement polymerization may have thermal necrosis effect [14]; (3) the methyl methacrylate monomer has cytotoxicity effect which may kill the surrounding cancer cells [15].

There are a few reports of PVP in C1 and C2 spines as they are less affected; however, they are the most technical challenging anatomic regions. Posteroanterior [16], posterolateral [17], transoral [18], and anterior retropharyngeal approach [19] have been reported to perform PVP in C1 or C2. We have only one C2 patient and we successfully used anterolateral lateral approach. One should keep in mind that if the patient is extremely overweight or has severe cervical disease to maintain cervical extended position, this approach is not applicable. The most common complication is cement leakage and our experience in preventing cement leakage is as follows: (1) preoperative CT scan is performed to check the integrity of the backside of the vertebrae body; (2) the procedure has to be performed under the monitor of imaging system and the injection should be stopped as soon as leakage happens; (3) the cement should be injected in a highly viscous form and must avoid too much dilution; (4) the volume of injected cement should be controlled since pain control is not proportional to lesion filling [20].

## 5. Conclusions

PVP procedure using anterolateral approach released the pain from the patients with multiple cervical metastases, which can improve their life quality and provide beneficial condition for further treatment.

## Figures and Tables

**Table 1:** Baseline demographics and disease characteristics.

Patient	Gender	Age	Primary cancer	Metastatic vertebrae	Adjusted Tokuhashi score	Tomita score	Preoperative NDI (%)	Preoperative VAS	Preoperative analgesics
1	Male	52	Lung	C2, C3	5	8	67	9	4
2	Male	69	Colon	C6, C7	9	7	58	8	3
3	Male	51	Lung	C3, C5, C6	6	7	62	8	4
4	Female	58	Lung	C4, C5	8	8	69	10	4
5	Female	47	Breast	C4, C6	7	7	49	9	3
6	Female	57	Breast	C5, C6, C7	10	6	55	5	1
7	Female	54	Lung	C6, C7	8	7	54	9	3
8	Male	61	Esophagus	C5, C6	6	8	43	7	2
9	Male	75	Lung	C3, C4, C5, C6	3	10	70	8	3

**Table 2:** VAS, analgesics, and NDI of patients at pre- and postoperative follow-up.

	Preoperative (*n* = 9)	Postoperative 3 days (*n* = 9)	Postoperative 3 months (*n* = 9)	Postoperative 6 months (*n* = 7)
VAS (±SD)	8.11 ± 1.45	2.22 ± 0.67^*∗∗∗*^	2.22 ± 0.67^*∗∗∗*^	3.14 ± 1.95^*∗∗∗*^
Analgesics (±SD)	3.00 ± 1.00	0.89 ± 0.78^*∗∗∗*^	1.00 ± 0.87^*∗∗∗*^	1.14 ± 0.69^*∗∗∗*^
NDI, % (±SD)	58.56 ± 9.28	40.89 ± 13.01^*∗∗*^	38.63 ± 14.80^*∗∗*^	37.86 ± 16.72^*∗∗*^

VAS: visual analog scale; NDI: neck disability index; SD: standard deviation.

^*∗∗*^ *P* value < 0.01 and ^*∗∗∗*^*P* value < 0.001 compared to preoperative follow-up.
